# Magnetogenetics as a promising tool for controlling cellular signaling pathways

**DOI:** 10.1186/s12951-024-02616-z

**Published:** 2024-06-10

**Authors:** Anastasiia A. Latypova, Alexey V. Yaremenko, Nadezhda A. Pechnikova, Artem S. Minin, Ilya V. Zubarev

**Affiliations:** 1 Institute of Future Biophysics, Dolgoprudny, 141701 Russia; 2 Center for Nanomedicine and Department of Anesthesiology, Brigham and Women’s Hospital, Harvard Medical School, Boston, MA 02115 USA; 3 Aristotle University of Thessaloniki, Thessaloniki, 54124 Greece; 4 Shemyakin-Ovchinnikov Institute of Bioorganic Chemistry of the Russian Academy of Sciences, Moscow, 117997 Russia; 5 Saint Petersburg Pasteur Institute, Saint Petersburg, 197101 Russia; 6 M.N. Mikheev Institute of Metal Physics of the Ural Branch of the Russian Academy of Sciences, Yekaterinburg, 620108 Russia; 7 Moscow Center for Advanced Studies, Moscow, 123592 Russia

**Keywords:** Magnetogenetics, Cell signaling, Mechanosensitivity, Mechanotransduction, Magnetic nanoparticles

## Abstract

Magnetogenetics emerges as a transformative approach for modulating cellular signaling pathways through the strategic application of magnetic fields and nanoparticles. This technique leverages the unique properties of magnetic nanoparticles (MNPs) to induce mechanical or thermal stimuli within cells, facilitating the activation of mechano- and thermosensitive proteins without the need for traditional ligand-receptor interactions. Unlike traditional modalities that often require invasive interventions and lack precision in targeting specific cellular functions, magnetogenetics offers a non-invasive alternative with the capacity for deep tissue penetration and the potential for targeting a broad spectrum of cellular processes. This review underscores magnetogenetics’ broad applicability, from steering stem cell differentiation to manipulating neuronal activity and immune responses, highlighting its potential in regenerative medicine, neuroscience, and cancer therapy. Furthermore, the review explores the challenges and future directions of magnetogenetics, including the development of genetically programmed magnetic nanoparticles and the integration of magnetic field-sensitive cells for in vivo applications. Magnetogenetics stands at the forefront of cellular manipulation technologies, offering novel insights into cellular signaling and opening new avenues for therapeutic interventions.

## Introduction

Cells sense the environment through biological signaling systems that affect gene expression. Some types of stimuli perceived by a cell are soluble low-molecular factors (hormones, growth factors), environment signals (extracellular matrix and adhesive molecules), antigens, and physical factors (mechanical stimuli, temperature change, or pH alteration) [1–4]. These signals regulate a wide range of cell activities, including survival, differentiation, migration, and proliferation [2, 5–9].

Controlling these essential cellular processes and uncovering their main participants remains one of the most important goals in the biological research field, especially for the needs of therapy. The vast majority of human diseases are known to be at least partially caused by deregulation and dysregulation of cell signaling pathways [10], including Alzheimer’s disease [11, 12] Parkinson’s disease [13, 14], cardiovascular diseases [14, 15], diabetic complications [16], and cancer [11, 14, 17–19]. Each discovery of the molecular basis for pathogenesis proposes new therapy targets or possible ways of disease control and diagnostics [19, 20]. Moreover, even non-pathogenesis-related signaling pathways may open new possibilities in some medical fields. Particularly, comprehending stem cell signaling holds significant promise for advancing regenerative medicine, and recent studies have suggested that cell signaling mechanisms could potentially bolster tissue regeneration [21–23].

The group of methods and approaches enabling cell signaling control and study is quite diverse and utilizes different principles of effect on a cell. However, the most classical and widespread approach consists of activation or inhibition of a protein function and consequent analysis of cell response. The perturbations may be caused by pharmaceutical treatment, such as specific kinase inhibitors, or by genetic treatment up- or down-regulating expression of a gene of interest [24, 25]. These two classical categories formed the basis of the omics approach to molecular perturbation and are expected to be the most common for a long time [26].

Pharmaceutical and genetic treatments may have limitations in certain circumstances. Cell signaling networks are highly complex and consist of chains, parallel pathways, and multiple intersections. The expression of many genes involved in producing and secreting antibodies, hormones, or growth factors follows a temporal pattern [27, 28]. Additionally, cells in vivo do not constantly secrete many proteins and instead require external stimuli for control [29]. The signal acquisition by the receptor, signal transmission to the nucleus, gene transcription, translation, and secretion into the extracellular space are processes that are tightly controlled at all stages [30]. The temporal capabilities of chemically inducible systems depend on the diffusion and half-life of inducing molecules, which impose restrictions on the action dynamics. Moreover, in the case of in vivo applications, invasive delivery of agonists may cause inflammation and other side effects.

To overcome these circumstances, several groups of methods that significantly broaden the horizons of cell signaling study and control have been developed recently. These methods have evolved to meet the current demands of the field such as reducing invasiveness, developing in vivo experiments, and applicability to complex systems such as neuronal signaling. The most widely known group of these methods is optogenetics, which uses light to report on and control signaling proteins in cells. It was shown, that optogenetics can provide both high temporal (at least microseconds) and spatial (at least microns) resolution [31]. So, the optogenetic approach has been applied to control various neuronal processes wirelessly and remotely in vivo [32–34], as well as to control cell signaling and epigenetic states [35, 36]. However, despite the increasing use of this powerful tool, some technical limitations still prevent it from becoming a universal solution for manipulating cellular activity. One of them is that optogenetics application in vivo requires an implant with a light source, leading to side effects of chronic surgery and laser-induced heating [37]. Another one is that optogenetics applications are limited by the light penetration depth [38]. Moreover, diffusion of the photoproteins and possible off-target effects during genetic modification might cause a decrease in the accuracy of gained results, even though several solutions to these problems have been suggested [39].

**Fig. 1:**
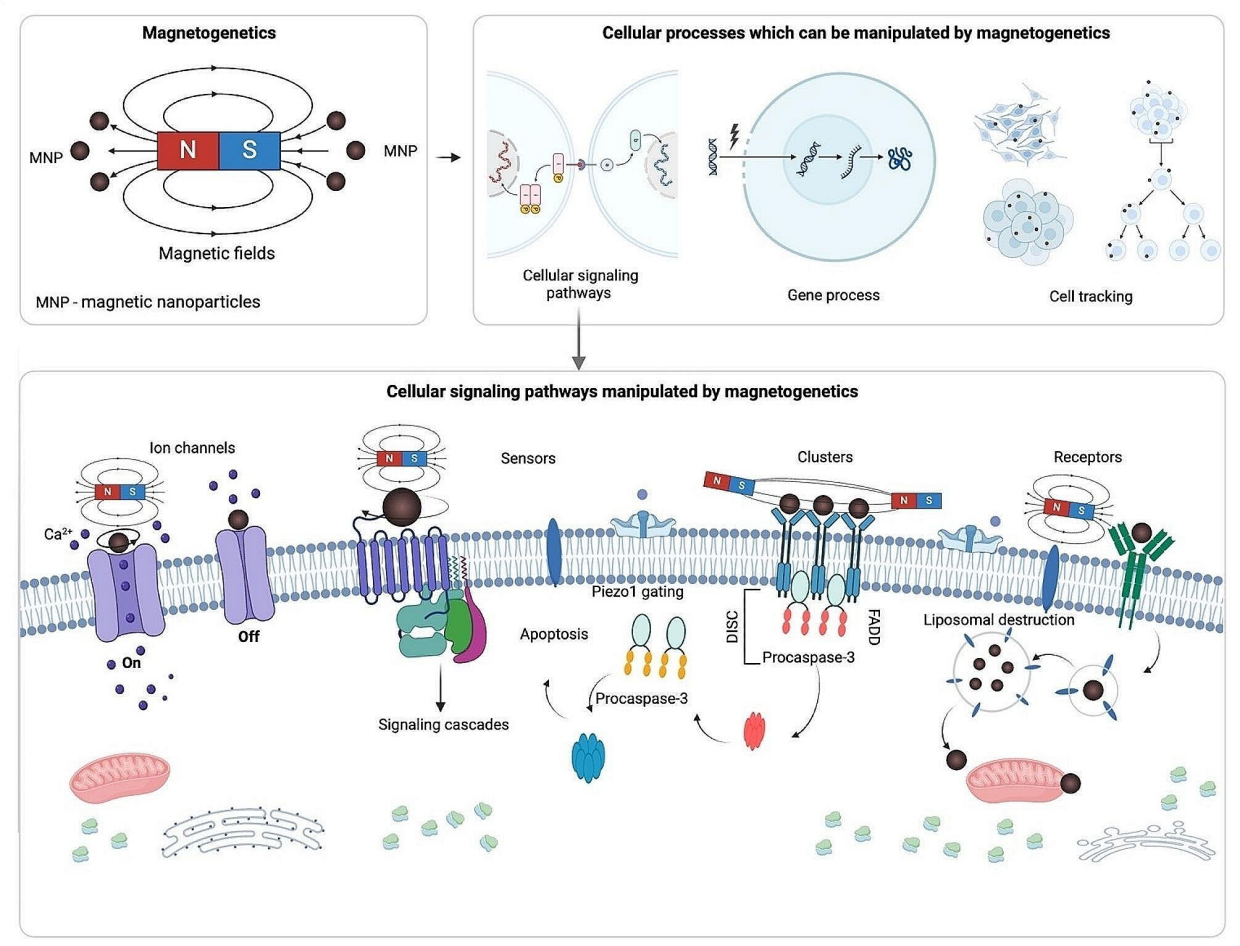
Exploring the potential of magnetogenetics in cellular modulation. This review outlines the current state of magnetogenetics as a versatile tool for the precise control of cellular signaling pathways. It details the application of magnetic fields and nanoparticles for the direct manipulation of ion channels, the activation of mechanosensitive sensors, the clustering of membrane proteins, and the targeting of cellular receptors. The review also addresses how magnetogenetics influences gene expression, orchestrates signaling cascades, and facilitates cell tracking. It presents the strategies for nanoparticle encapsulation and discusses their effects on cellular processes such as apoptosis and liposomal destruction. The potential of magnetogenetics is posited to be transformative for precision medicine and bioengineering, setting the stage for future innovations [65–67]

An alternative approach that is not restricted by these limitations is magnetogenetics. This approach was introduced in a commentary [40] on a study conducted by Pralle et al. in 2010 [41] and utilizes magnetic fields applied to targeted magnetic nanoparticles (MNPs) to manipulate various cellular processes (Fig. 1). A key feature of the magnetogenetic approach is that the interaction between MNPs and the magnetic field provides competitive advantages and applications. Depending on the magnetic field characteristics, different types of MNPs can heat their targets or apply a mechanical force to move them, e.g., rotate, pull, push, or cluster (Fig. 1). This mechanical action is different from ligand-induced interaction and can be useful for manipulation of mechanically-induced cell signaling. In an organism mechanical stress does not only affect specialized mechanosensitive cells but also is a major regulator of various cellular processes influencing the development and homeostasis of tissues and whole organisms [42, 43]. Shear stress, tension, and compression affect the behavior of both individual cells and tissues, organs and systems. A breach in normal mechanotransduction regulation has been associated with severe diseases, including developmental defects, cardiovascular diseases, and cancer [42]. In prior studies, magnetic actuators have been applied to mechanically act on a cell to control cell fate for cancer therapy [44–48] and especially immunotherapy of cancer [49, 50]. Moreover, perhaps mechanical cell signaling control through magnetogenetics has found even greater use in regenerative medicine, enabling stem cell activation [51], inducing differentiation [51, 52], and driving tissue formation [53]. Besides the mechanical effects of magnetogenetics, high-frequency magnetic fields enable heating MNPs, thus they can activate thermosensitive ion channels [54], and initiate heat-responsive promoters for the needs of cancer therapy [49] and regenerative medicine [55].

Bridging the exploration of magnetogenetics from its foundational applications in manipulating cellular processes through mechanical and thermal stimulations, the technique’s versatility extends beyond these initial capabilities. Many receptors traditionally considered non-mechanosensitive may be activated by clustering, rotation, or conformational changes – actions that MNPs are capable of performing [22, 56–60]. Moreover, the activation of such receptors by the magnetogenetic approach addresses some challenges of analogous methods. Compared to light, magnetic fields do not interact with living tissue, are not absorbed, and do not weaken. Therefore, if the magnet construction enables a sufficient field at a distance [61–63], there is no need for surgical implantation of the source of the magnetic field into the organ of interest. Additionally, the frequency and duration of magnetic stimulation can be easily controlled and spatially delivered [64], allowing the pattern of action on the cells to be dynamically changed. These and other features of magnetogenetics make it a promising tool for manipulating cellular signaling pathways in vitro and in vivo, for fundamental studies and therapeutic applications.

The objective of this review is to elucidate magnetogenetics as an innovative and promising strategy for the modulation and elucidation of cellular signaling mechanisms. In this paper we overview the technological nuances and methodologies underpinning magnetogenetic interventions, focusing on the diverse effects these approaches can have on cellular signaling pathways. Our discussion includes a broad spectrum of cellular targets, differentiating between those responsive to thermomechanical stimuli alone and those necessitating the concomitant presence of an activating ligand. Furthermore, we critically examine the spectrum of applications that magnetogenetics has already found across various domains, as well as its potential future contributions to the field. Through a comprehensive exploration, we aim to highlight the significant advantages magnetogenetics offers over conventional methods, particularly in terms of its non-invasive nature, the precision of spatial and temporal control, and its versatility in addressing complex biological questions and therapeutic challenges. This review also underscores the transformative potential of magnetogenetics in advancing our understanding of cellular signaling pathways and in pioneering novel therapeutic approaches, thereby opening new avenues for research and application in both fundamental and clinical sciences.

## Basic principles of magnetogenetics

**Fig. 2:**
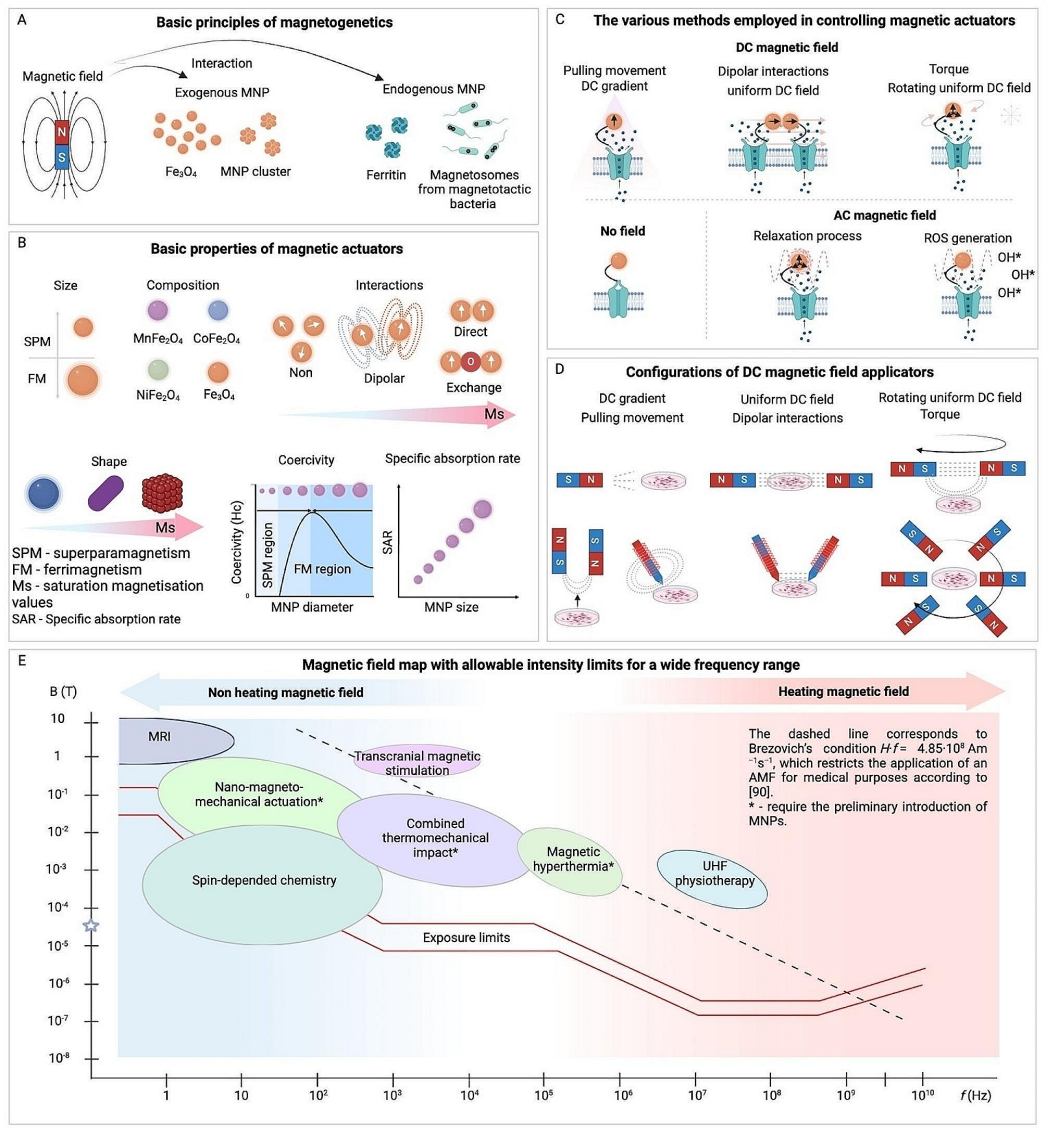
Overview of magnetogenetic principles and applications. (**A**) Schematic of the basic principles of magnetogenetics showing the interaction of a magnetic field with both exogenous and endogenous magnetic nanoparticles (MNPs). (**B**) Properties of magnetic actuators classified according to size, composition, and interaction types with magnetic fields, illustrated with examples of superparamagnetism (SPM), ferrimagnetism (FM), and varying coercivity and saturation magnetization (Ms) based on MNP diameter [65, 81]. (**C**) Methods to control magnetic actuators with no field, DC magnetic field, and AC magnetic field, showing the mechanical effects (e.g., pulling movement, torque, relaxation process, and ROS generation) [65]. (**D**) Different configurations of DC magnetic field applicators and their corresponding magnetic orientations and interactions (gradient, uniform, rotating uniform) [65]. (**E**) A magnetic field map highlighting non-heating and heating magnetic field intensity limits across a wide frequency range [82]

The fundamental components of magnetogenetics are a magnetic field and modified MNPs that are applied externally to interact with a cellular target. These elements’ characteristics determine their effects on the targets, so both magnetic fields and MNPs need optimal configurations and properties to manipulate cellular processes in vitro or in vivo effectively. Additionally, depending on their characteristics, there are several different types of action on target molecules, ranging from thermal effects to mechanical force applications. A summary of various methodological features of magnetogenetic approaches is presented in Table 1; Fig. 2.

**Table 1:** Overview of magnetogenetic techniques: configurations and applications. MNP – magnetic nanoparticle; N/A – data not available; Ms – saturation magnetization is the maximum magnetic moment per unit volume for a magnetic material. Ms ultimately determines the force with which a particle moves along a magnetic field gradient; Abs – antibodies; DR4 Ab – death receptor 4 antibody; hASC – human adipose-derived stem cells; TREK-1 – potassium channel subfamily K member 2; hMSC - human mesenchymal stem cells; hBMSC - human bone marrow-derived mesenchymal stem cells; TRPV1 - transient receptor potential vanilloid 1; CCK2R – cholecystokinin 2 receptor; αGFP – nanobody against monomeric enhanced green fluorescent protein (mEGFP); TNFα – tumor necrosis factor alpha; SBP – streptavidin binding peptide; TIAM – catalytically active domain of T-cell lymphoma invasion and metastasis-inducing protein; PMAO – poly(maleic anhydride-alt-1-octadecene); DSPE-PEG – 1,2-distearoyl-sn-glycero-3-phosphoethanolamine-N-[methoxy(polyethylene glycol)]; H – magnetic field strength; *f* – frequency of alternating magnetic field.

	Magnetogenetic task	MNP composition	MNP size	MNP Ms	MNP surface modification	Applied magnetic field (H and f)	Principle of action	Ref.
Permanent magnetic field	Actin filament manipulation in vitro	Fe_3_O_4_	10 nm	N/A	Streptavidin	N/A	Shear force	[68]
	Rapid spatial reorganization of proteins captured to the nanoparticle surface	Engineered ferritin	20 nm	87 emu/g	αGFP; TNFα; SBP; TIAM	N/A	Shear force	[69]
	Acute neural stimulation in constant gradient	Fe_3_O_4_	100 nm	40 emu/g	Starch	N/A	Shear force	[70]
	Magnetically controlled DR4 apoptosis induction	Zn_0.4_Fe_2.6_O	15 nm	161 emu/g	DR4 Abs; doxorubicin	N/A	Cluster of MNPs assembly	[44]
	Piezo1 receptor stimulation in cell culture	Fe_3_O_4_	75 nm	N/A	A-bungarotoxin	40 mT –	Shear force	[71]
	Polarization of stem cells	Fe_3_O_4_	N/A	70 emu/g	SiO_2_ – actin binding peptide	0–3.81 mT –	Assembly of MNPs clusters	[72]
Low-frequency magnetic field	External manipulation of activin receptor type IIA in hASC	Fe_3_O_4_	250 nm	N/A	Dextran@Abs	25 mT 1 Hz	Oscillating shear force	[51]
	Activation of TREK-1 in hMSC	Fe_3_O_4_	300 nm	N/A	Dextran@Abs	25 mT 1 Hz	Oscillating shear force	[73]
	Osteogenic differentiation of bone marrow-derived hMSC	Fe_3_O_4_	250 nm	N/A	Dextran@Abs	60 − 120 mT 1 Hz	Oscillating shear force	[74]
	hBMSC differentiation towards a smooth muscle cell lineage	Fe_3_O_4_	250 nm	N/A	Dextran@Abs	60 − 120 mT 1 Hz	Oscillating shear force	[75]
	Magnetomechanical neuronal stimulation with nanodiscs	Fe_3_O_4_	280 nm	110 emu/g	PMAO	50 mT 10 Hz	Oscillating shear force	[76]
	Chymotrypsin catalytic activity change	Fe_3_O_4_	25 nm	N/A	Au	5–250 mT 16–500 Hz	MNPs rotation	[77]
High-frequency magnetic field	Thermal TRPV1 activation in neurons	MnFe_2_O_4_	6 nm	~ 70 emu/g	Streptavidin	0.84 mT 40 MHz	Heating	[41]
	Magnetic activation of neurons, heat-sensitized by expressing TRPV1	CoFe_2_O_4_@MnFe_2_O_4_	10 nm	~ 70 emu/g	Neutravidin	46 mT 412.5 kHz	Heating	[78]
	Stimulation of heat sensitive TRPA1-A in fly neurons	Fe_3_O_4_@CoFe_2_O_4_; Fe_3_O_4_	15 nm; 40 nm	~ 70 emu/g	DSPE-PEG	10–80 mT 0.05–5 MHz	Heating	[79]
	Cell death activation through internalization of CCK2R	Fe_3_O_4_	10 nm	80 emu/g	Gastrine	24–40 mT 275 kHz	Heating	[80]

### Exogenous magnetic nanoparticles in magnetogenetics

Exogenous MNPs are one of two main components of magnetogenetics, acting as indispensable actuators for the modulation of cellular activity. These MNPs are selectively engineered, with their efficacy contingent upon a precise set of parameters including their size, composition, magnetic properties, and surface coating to ensure compatibility with magnetogenetic techniques (Fig. 2A-B) [67, 81, 82].

For optimal functionality, a magnetogenetic actuator must exhibit a large magnetic moment to exert the necessary mechanical force [83], possess a high coercivity for particle rotation [84], or have the capacity to generate heat for localized thermal interventions [85]. The magnetic characteristics of these particles are defined by their specific iron-based compounds, primarily ferrites, which may be combined with additional elements like cobalt, manganese, or zinc to enhance their properties. The safety profile of these MNPs is a crucial consideration, as evidenced by extensive research on their biocompatibility, including studies on magnetic hyperthermia in cell cultures [86] and animal models [87]. Generally, these studies suggest that iron compound MNPs are non-toxic at low concentrations [88–90]. However, it is important to note that the toxicity can be significantly influenced by the particles’ surface modifications, a factor that necessitates careful consideration in their design for magnetogenetic applications [91].

In magnetogenetic applications, the size and shape of particles are also crucial. While these particles are typically spherical, there is a wide range of sizes (Fig. 2B). The desired localization of nanoparticles, whether on the cell surface or intracellularly, determines their optimal size [92]. While surface-bound nanoparticles can vary in size, with some successfully binding to receptors like Piezo1 even at diameters up to 200 nm [93], for intracellular action, smaller nanoparticles are preferred to facilitate cellular entry and movement within the cytoplasm. The optimal size of MNPs for magnetogenetic applications is a delicate balance, typically ranging from 20 to 25 nanometers [94] in diameter to enable efficient navigation through the cytoplasm and exertion of mechanical forces without causing cellular damage. These dimensions ensure magnetic responsiveness and compatibility with in vivo mechanical forces, which are generally within the piconewton range [65, 94]. Notably, 20 nm magnetite MNPs have been shown to activate intracellular signaling by exerting mechanical forces between 0.2 and 38.9 piconewtons per particle [95]. Also it was demonstrated, that MNPs with a diameter under 50 nm could be manipulated in living cells with magnetic forces in the femtonewton range (fN or hundreds of fN) rather than the piconewton range under the viscoelastic conditions of the cytoplasm [96]. Furthermore, the clustering of MNPs can amplify the exerted mechanical force [97], potentially allowing for magneto-controlled rearrangements of cellular components [70].

Beyond the physical dimensions, the magnetic properties of nanoparticles such specifically saturation magnetization (Ms), coercive force (Hc), and specific absorption rate (SAR) also play crucial roles in their effects on a target. Widely used in magnetogenetics Fe_3_O_4_ nanoparticles typically exhibit an average saturation magnetization of around 80 emu/g, which can increase up to 140 emu/g. However, a higher magnetization does not necessarily mean a stronger force application as the exerted force does not scale linearly with Ms. The clustering of nanoparticles can also significantly affect the force exerted, leading to nonlinear increases in force that must be considered when predicting the effects of MNPs on targeted cells or tissues [97].

In addition to physical characteristics, chemical surface modifications also play a significant role in the functionality of MNPs. To enhance surface properties and biocompatibility, MNPs are commonly functionalized using EDC conjugation and biotin-streptavidin binding. These methods allow for the attachment of various biologically active molecules and antibodies to the nanoparticles, increasing specificity for membrane receptors​ [98–101]​. This enhanced specificity enables MNPs to precisely target membrane proteins, thereby effectively modulating cellular pathways. However, ensuring compatibility between exogenous MNPs and targeted signaling pathways necessitates meticulous design. This design must consider not only the spatial and temporal aspects but also how these factors interact with external magnetic fields. To address it, established methods from magnetic hyperthermia are often adapted to magneto genetics, such as modifying nanoparticles with antibodies for targeted delivery [101, 102]. Additionally, innovative approaches include using modified ferritin as a magnetic core and subsequent GFP modification for visualization [69].

To enhance the effectiveness of MNPs in magnetogenetics, rational design techniques could also be employed to optimize their synthesis and modification processes. These techniques were successfully applied in developing biosensors, contrast agents, and hyperthermia agents, demonstrating their significant promise for future magnetogenetic applications [100–102]​​. By using targeting molecules such as actin-binding proteins and antibodies, these nanoparticles ensured selective and precise binding to target proteins, allowing specific localization down to individual amino acids [101]. Furthermore, it is essential to design nanoparticles that can accurately respond to external magnetic fields, ensuring precise spatial targeting and timing. So, future advancements in the rational design and engineering of magnetic nanoparticles are expected to significantly enhance the precision and efficacy of magnetogenetics, opening new avenues for biomedical research and therapeutic applications [100–102]​. Thus, the successful application of exogenous MNPs in magnetogenetics hinged on a complex interplay of their physical dimensions, magnetic properties, and chemical surface modifications. The precise engineering of these particles enabled targeted modulation of cellular activity, offering vast potential for biomedical research and therapeutic interventions.

### Endogenous magnetic nanoparticles in magnetogenetics

Beyond the use of exogenous magnetic nanoparticles, which are primarily used in vitro, magnetogenetic research has explored the potential of endogenous magnetic actuators as alternative mechanisms for the stimulation of cell signaling (Fig. 2A-B). Endogenous actuators circumvent the in vivo limitations associated with exogenous nanoparticles, which include the challenge of delivering these particles through tissue to reach intracellular targets. This challenge often compromises the non-invasive nature and deep tissue penetration capabilities inherent to magnetogenetics. To address this, strategies such as optimizing culture conditions or implementing genetic programs that induce the expression of genes responsible for nanoparticle formation within target cells have been proposed [103–108]. These approaches enable the development of intracellular, genetically encoded magnetic actuators, thereby enhancing the feasibility and effectiveness of applying magnetic fields for in vivo applications. This innovation opens up new avenues for non-invasive cellular manipulation, leveraging the unique advantages of magnetogenetics in living organisms.

Among the proteins of natural origin, primary attention is paid to ferritin, a protein sequestering and holding theoretically up to 4500 iron atoms in its inner cavities [109]. Even though the exact organization of iron inside the ferritin cavities has not been revealed yet, magnetic behavior of ferritin has garnered significant attention in magnetogenetic studies. Ferritin-based constructions with a 7 nm magnetic core have been demonstrated to produce mechanical force in the fN scale [69]. Additionally, it was presented that micrometric genetically modified ferritin clusters can produce a high mechanical force of about 10 pN (cluster formed of about 10^5^ ferritin molecules) [75]. In some works on dried and frozen ferritin, it was described as ferromagnetic or superparamagnetic (although the measured saturation magnetization is 0.5 emu/g compared to 80–100 emu/g for Fe_3_O_4_ nanoparticles) [110, 111], while the ferritin molecules in water solution show antiferromagnetic properties [112]. Moreover, ferritin showed no measurable local or bulk heating upon exposure to an alternating magnetic field (AMF) [112].

However, despite the current uncertainties in characterization of ferritin molecules, various ferritin-based magnetogenetic methods have been applied to manipulate cellular processes. For instance, GFP-tagged ferritin fusion protein tethered to TRPV1 was presented for non-invasive, temporal activation or inhibition of neuronal activity in vivo and for the study of central nervous system control of glucose homeostasis and feeding in mice [113]. In addition, ferritin-dependent non-invasive electromagnetic control over ion channels revealed a negative effect of maternal hyperthermia on fetal development [114]. Generally, one of the most frequent targets of ferritin-based magnetogenetics is transient receptor potential vanilloid channels (TRPV). Engineered ferritin tethering has been employed to activate TRPV in cell cultures, mice, and chick embryos [114–117]. However, even though TRPV is intrinsically temperature and mechanically sensitive and some early works proposed that the interaction between magnetic fields and ferritin produces heat or mechanical stimuli that directly activate TRPV [113, 117], in recent publications another explanation for this phenomenon was suggested. Several studies supposed that an alternating magnetic field triggers the dissociation of iron from the ferritin and thus generates reactive oxygen species (ROS), short-chain fatty acids, and oxidized lipids, which activate the TRPV [115, 118]. Consequently, the observed effects of ferritin may not be the result of TRPV activation, since ROS activate a variety of cellular targets that influence cell functioning. As a matter of fact, many of the parameters associated with ferritin as a magnetogenetic actuator are still unknown. The scientific community has expressed some doubts about ferritin-based magnetogenetic systems that can induce neuronal activation. Due to the inability to successfully reproduce earlier results using these constructs, three different research teams doubted the previous magnetogenetics conclusions [119–121]. Thus, further research into the precise mechanisms of ferritin-based magnetogenetics will undoubtedly contribute to the growing understanding of this intriguing area of study.

In parallel, magnetotactic bacteria (MTB) present another promising avenue for endogenous magnetic actuation. MTBs naturally form magnetosomes, magnetic particles enveloped in bilipid membranes, controlled by specific proteins that dictate the crystallization of magnetic nanoparticles [122]. These bacterial magnetosomes are susceptible to magnetic fields ∼0.5 Gauss = 0.05 mT [123] and have magnetic moments around 2·10^–16^ A/m^2^ in magnetic fields below 23 mT [124]. The diameter of most magnetosome crystals ranges from 35 to 120 nm [125], alongside a size range conducive to cellular manipulation, represent a compelling alternative for magnetogenetic applications. Efforts to express MTB proteins in eukaryotic cells [104–107, 125], or even create chimeras between ferritin and MTB proteins [126], have shown potential, suggesting that the field of magnetogenetics may soon expand significantly with these biological innovations.

### Magnetic field and action on a target in magnetogenetics

A fundamental advantage of magnetogenetics lies in its capacity to precisely regulate external magnetic fields, enabling the application of varied stresses and forces on biological systems [65]. This leads to the necessity for a device capable of generating a magnetic field to influence particles within a biological media, marking the final component essential to the magnetogenetic methodology (Fig. 2C-D). Research in this area is distinguished by the type of magnetic field utilized, ranging from permanent magnets that create constant magnetic fields to systems that generate low-frequency and high-frequency fields. The complexity and cost associated with these magnetic systems increase alongside their size and field strength. For cell culture experiments, simpler magnetic setups are sufficient, but more sophisticated systems are needed for conducting studies on animals, including larger species [127] and potentially humans [128]. When high-gradient fields are required, opting for existing magnetic resonance imaging (MRI) equipment often emerges as a more feasible approach than the development of new devices. However, this option also has challenges, primarily due to the limited gradient achievable within an MRI’s operational zone and the higher gradients found outside of it, potentially complicating the design of experiments (Fig. 2E).

Focusing on systems employing permanent magnets, these setups enable particles to migrate towards areas of increasing magnetic field gradients, thereby exerting a constant pressure that is directly proportional to the gradient. Constructing a magnetic field system with permanent magnets is relatively straightforward [129], with NdFeB magnets being widely available in various shapes and sizes at reasonable prices. The key benefits of using such permanent magnet systems include their operational independence from external power sources and the absence of the need for complex control mechanisms. However, a notable limitation of these systems is their inability to be simply switched off; the only way to halt their influence on a biological object is to physically remove the object from the system’s coverage area. Another critical limitation of the permanent magnetic field is the complicity in manipulating the magnetic field’s gradient, which is usually more significant than the strength of magnets [130]. To achieve appropriate results typical gradients should fall within the range of 10–100 T/m on a millimeter scale [70]. This gradient acts as the principal factor determining the force exerted on a particle. So, to achieve such gradients numerous devices were presented including magnetic tweezers which offer a solution with their highly localized and precise impact at the cellular level, capable of producing significantly larger gradients, up to 1·10^9^ T/m on a micrometer scale, showcasing the diverse capabilities and considerations required in the design and implementation of magnetic systems in magnetogenetics.

Expanding upon the use of permanent magnets, introducing an actuator into a permanent magnet system and moving it can create a low-frequency alternating magnetic field [75]. This approach, often more practical than employing large solenoids with complex electronics, enables the achievement of frequencies ranging from tens to hundreds of Hz. These systems maintain field gradients of several T/m and field strengths of tens of mT. Moreover, the development of such devices is not restricted by using of permanent magnets and similar low-frequency systems can also be built using solenoids [76]. The advantages of this technique are that at these frequencies thermal heating is minimal, and the primary effect is mechanical. The movement of particles in a single axis or their rotation around an object introduces pulsating pressure that varies with the magnetic field gradient, with the force depending on the system’s rotation speed. This nuanced approach to generating and manipulating magnetic fields illustrates the versatility and precision available in magnetogenetics, offering a wide array of possibilities for exploring cellular processes.

As the frequency of magnetic field oscillation increases to hundreds of kHz or even MHz, the nature of the forces exerted on particles and their interaction with target proteins transforms. This shift from mechanical movement to heating has spurred the development of various high-frequency devices capable of heating magnetic nanoparticles, some of which are commercially available [131] and designed for use in animal studies [132]. Despite the prevalence of research on magnetic hyperthermia for cell destruction, only a select few studies have explored the potential of such high-frequency fields in controlling cell signaling through the activation of thermosensitive channels [133].

Moreover, the controlled clustering and dimerization of membrane receptors or the modification of specific molecules’ intracellular distribution represent additional strategies for remotely controlling cellular signaling with magnetic actuators [56, 58, 60]. When MNPs are tethered to membrane receptors, the applied magnetic field can induce clustering or oligomerization, thereby activating downstream signaling pathways. This concept has led to groundbreaking research, demonstrating that heat, mechanical tension, or spatial rearrangement facilitated by magnetic fields can be instrumental in exploring fundamental cell signaling processes, such as generating action potentials in brain cells [54] or influencing cell migration and differentiation [69].

Building on these innovative approaches, the future of magnetogenetics holds exciting possibilities with the potential incorporation of novel materials and mechanisms. One promising avenue involves multiferroic materials, which can induce changes in electric polarization when exposed to a magnetic field [134]. These materials have already demonstrated their magnetic properties in applications such as drug release in vitro to induce apoptosis in carcinoma cell lines [135]. So, the future exploration of their electric polarization effects in biological processes could open new frontiers in magnetogenetics. Additionally, materials that affect photon polarization under a magnetic field [136] could potentially be integrated with optogenetics [137], creating innovative methods for manipulating cellular functions with high precision.

Another promising direction for the development of magnetogenetics is the utilization of natural magnetoreception mechanisms. Although the biomolecular details of this process are not yet fully understood, recent studies suggest that magnetoreception in animals may rely on mechanisms involving biogenic magnetite and cryptochromes. For instance, some species form chains of intracellular magnetite crystals, which act as internal compasses, aligning with the Earth’s magnetic field and potentially interacting with mechanosensitive channels to transduce magnetic information into cellular signals [138, 139]. Additionally, cryptochromes, which are flavoproteins found in various organisms, might play a crucial role in light-dependent magnetoreception through the formation of radical pairs influenced by magnetic fields​ [140, 141]​. Leveraging these natural mechanisms could significantly simplify magnetogenetic techniques, much like how the use of natural photosensitive proteins has revolutionized optogenetics into a universal tool. These advancements underscore the vast potential of magnetogenetics to enhance biomedical applications and inspire future innovations in the field [139]​.

### Mechanical forces and temperature quantification at the nanoscale

Magnetogenetics primarily relies on cellular responses to demonstrate the effects of applied magnetothermal or magnetomechanical actions, rather than directly measuring local temperature or mechanical force within the cell. Ideally, influencing the cell and monitoring it simultaneously would provide more direct insights, but this approach can be complicated and because of this has not been extensively implemented in magnetogenetics studies. Instead, usually researchers measure the effects indirectly through various indicators such as changes in cell activity [142], alterations in cell type [51], or induction of cell death [44]. Additionally, to assess the impact of magnetic stimuli shifts in metabolism [113] and behavioral changes in animal models [143] can be used.

However, to fully understand the mechanisms involved, characterizing the mechanical force or heating generated by a magnetic actuator is crucial. This is particularly important as the properties of MNPs enabling local heating or mechanical force application may differ in vitro and in vivo compared to *as-prepared* colloids [144, 145]. One approach involves measuring the direct effects of MNPs in media resembling the cytoplasm. For example, the mTorquer system’s torque force has been measured using Stokes’ law in viscous media by visualizing a fluorescent probe attached to its surface [59]. Various theoretical calculations and numerical simulations also predict the local effect of magnetic activation in the intracellular environment for both thermal [144] and mechanical effects [146]. Additionally, multiple methods of direct measurement in vitro and in vivo are available.

Accurate measurement of MNP temperature in vivo has been achieved using the ratio of the fifth and third harmonics of the magnetization generated by magnetic nanoparticles in a sinusoidal field. This method allows for precise temperature quantification at the nanoscale, providing crucial data for understanding the thermal effects of magnetogenetic applications [147]. Fluorescent dyes are also employed to measure intracellular temperature, either introduced into the cell from the outside [148] or expressed internally. For instance, a study on magnetic hyperthermia developed a GFP nanothermosensor enabling direct temperature measurement within a cell, providing real-time thermal data that are critical for controlling and optimizing hyperthermia treatments [149]. Additionally, various nanoscale thermometers potentially applicable within and beyond magnetogenetics have been reviewed, highlighting their diverse applications and the technological advancements in the field of intracellular measurement [150].

Approaches for measuring various rheological intracellular parameters using magnetic nanoparticles are also available, offering insights into the mechanical environment within cells. For instance, magnetic nanowires coated with gold can measure the viscosity of cells by rotating these rods with a magnetic field and analyzing the plasmon resonance spectrum on their coating. This technique provides detailed information about the viscoelastic properties of the cellular cytoplasm, which is essential for understanding how cells respond to mechanical stimuli [151]. Another method involves measuring local pH in the cell using nanoparticles bound to a fluorescent dye targeted to specific parts of the cell. This allows for precise monitoring of pH changes within distinct cellular compartments, contributing to a better understanding of cellular metabolism and signaling [152].

These advanced measurement techniques are crucial for fine-tuning the characteristics of magnetic fields and nanoparticles to optimize their effectiveness in targeted applications. By understanding the magnitude of mechanical forces and local heating sufficient to activate specific signaling pathways, more precise modulation of cellular activity can be achieved. This comprehensive understanding enhances the potential of magnetogenetics in biomedical research and therapeutic interventions, paving the way for innovative treatments and applications in various fields of medicine. Fine-tuning these parameters not only optimizes the efficacy of magnetogenetics but also expands its applicability, making it a versatile tool for future scientific advancements.

## Application of magnetogenetics for mechano- and thermosensitivity associated pathways activation

Mechanotransduction is the process through which cells translate mechanical stimuli into biochemical signals, a fundamental form of sensory transmission believed to be among the first to evolve in living organisms, spanning across the Eukarya, Bacteria, and Archaea domains. This widespread presence across diverse life forms underscores the primordial and essential nature of the ability to sense and respond to mechanical changes in the environment [153, 154]. Similarly, thermosensitivity is another basic sensory perception that organisms possess, allowing them to respond to temperature variations. This is achieved through various mechanisms, including “molecular thermometers” that are soluble in the cell’s cytoplasm and thermosensitive transmembrane ion channels that directly affect the cell’s membrane potential in response to temperature changes [155, 156]. Both mechanotransduction and thermosensitivity represent critical modalities by which organisms interact with their surroundings, facilitating adaptation and survival through a complex interplay of physical and biochemical responses. So, exploring the foundational mechanisms of these modalities is vital for scientific advancement, and one of the innovative approaches that allow realizing this exploration can be magnetogenetics.

Magnetogenetics offers a powerful tool for activating both mechano- and thermosensitive molecules, along with their downstream pathways. This technique primarily targets molecular structures that can be influenced mechanically or thermally, making it ideal for investigating the principles of mechanotransduction. Examples of such molecular structures include specialized membrane proteins like ion channels and cell surface receptors, as well as cell junction molecules, focal adhesion molecules, and specific cytoskeletal proteins. These proteins play a crucial role in sensing and transmitting mechanical stress within cells. When exposed to stimuli such as cell membrane stretch, pressure, or alterations in the external environment’s mechanical properties, these mechanosensitive proteins and their complexes can trigger downstream signal transduction [157]. The development of magnetogenetics to control these processes—naturally induced by mechanical or thermal events—highlights its significance. Additionally, a key advantage of using magnetogenetics lies in its ability to initiate signaling pathways through receptor dimerization or changes in conformation.

### Activation of mechano- and thermosensitive ion channels via magnetogenetics

A crucial role in these signaling pathways plays mechano- and thermosensitive channels which convert mechanical or temperature stimuli into electrochemical gradients by adjusting their opening rate in response to physical activation [2, 158–160]. These channels are integrated into a variety of physiological functions and can be involved in critical health issues, such as respiratory [161], and cardiovascular [162] diseases, neurological disorders [161], inflammatory bowel disease, and the mechanisms of pain [163], underscoring the therapeutic potential of understanding and manipulating these channels. Magnetogenetics stands out not only for its ability to influence these processes, naturally triggered by mechanical or thermal stimuli, but also for its precision in initiating signaling pathways. This precision is achieved through mechanisms like receptor dimerization or conformational changes, thus broadening our comprehension of cellular signaling mechanisms.

**Fig. 3:**
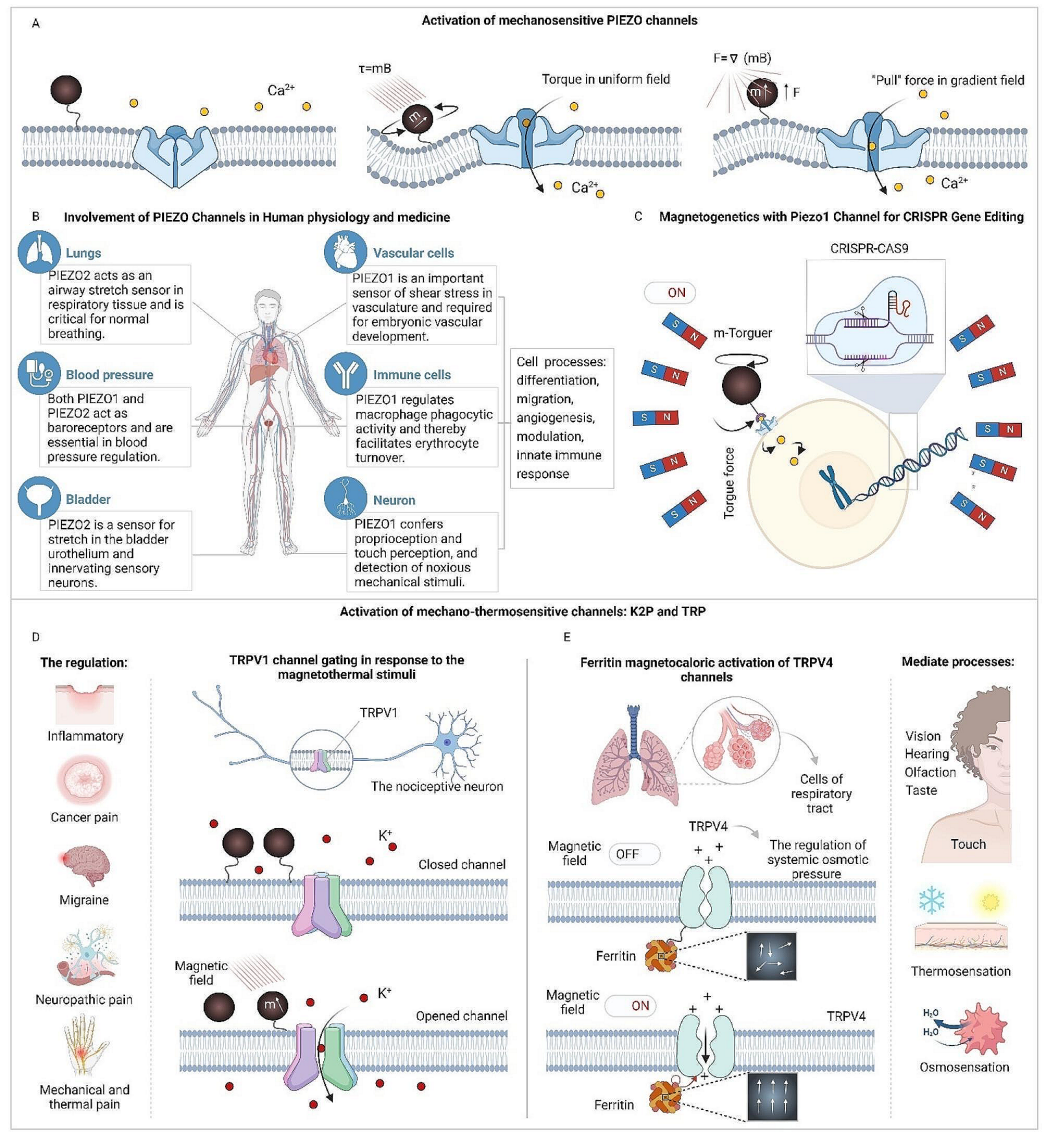
Utilizing magnetogenetics to activate mechano- and thermosensitive pathways. (**A**) Magnetic forces applied to the membrane region near mechanosensitive channels PIEZO1/PIEZO2 can activate them. This activation can occur through torque exerted by uniform magnetic fields or by pulling membrane sections tethered to nanoparticles in magnetic field gradients [164]. (**B** The role of PIEZO channels in human physiology and medical applications [165, 166]. (**C**) Using the Piezo1 channel in magnetogenetics for CRISPR gene editing [93]. (**D-E**) Stimulation of mechano-thermosensitive channels: K2P and TRP. (**D**) (Left) Information about that TRPV1 regulates processes such as inflammatory, pain from different etiology, migraine. (Right) Thermally gated ion channels, like TRPV1, activate in response to the hysteretic heating of nearby magnetic nanoparticles when exposed to magnetic fields alternating at frequencies exceeding 100 kHz [164]. (**E**) Activation of TRPV4 channels through ferritin magnetocalorics [142]. (Left) The diagram illustrates the mechanism by which the magnetocaloric effect in ferritin can trigger nearby temperature-sensitive ion channels like TRPV4. When a magnetic field is applied, it aligns the magnetic moments within paramagnetic ferritin nanoparticles, thereby lowering the magnetic entropy. This reduction in magnetic entropy produces heat (**Q**) through the magnetocaloric effect, which in turn can activate a nearby temperature-sensitive ion channel. Although ferritin had been represented as a paramagnet here, the computations remain the same for superparamagnetic particles. (Right) TRPV4 channels indirectly participate in various processes [158–160]

Building on this foundation, the Piezo channel family, including Piezo1 and Piezo2, exemplifies the intricate role of mechano-sensitive channels in regulating physiological processes in mammals (Fig. 3A) [167]. Acting as nonselective cation pores, Piezo channels respond to a variety of mechanical stimuli including laminar flow, cellular compression, membrane tension, cell swelling, and ultrasound [168, 169]. In addition, these channels play key roles not only in mechanosensory functions but also in essential developmental and regulatory processes such as stem cell differentiation, cell migration, angiogenesis, and the innate immune response (Fig. 3B) [2]. The ability of Piezo channels to conduct both monovalent (such as Na^+^ and K^+^) and divalent (such as Ca^2+^ and Mg^2+^) cations when activated underscores their function as excitatory channels, leading to membrane depolarization. In particular, the Ca^2+^ influx facilitated by Piezo channels triggers further intracellular Ca^2+^ signaling pathways, critical for processes like the mechanosensitive lineage choice in neural stem cells [170]. The integration of magnetogenetics with Piezo channels, particularly through magnetomechanical activation of Piezo1 using magnetic fields and MNPs [71], showcases a novel intersection of technology and biology. For instance, a magnetic torque actuator functionalized with an anti-Myc antibody was constructed to control neuronal activity by Myc-tagged Piezo1 activation in mice. When the magnetic field was applied, an increase in intracellular calcium influx was observed, whereas control groups showed no calcium responses [59]. Moreover, this approach has demonstrated the potential for remote control of neuronal activity and gene editing via the CRISPR system (Fig. 3C), prompted by magnetomechanical stimulation and consequent Ca^2+^ signaling. Such advancements demonstrated abilities to edit the target genome in vitro and large-scale brain phantom, mimicking the in vivo environment [93].

Beyond the Piezo family, a significant body of research has been dedicated to exploring how magnetic particles can specifically target other mechano-thermosensitive channel families, such as the two-pore potassium (K2P) and Transient Receptor Potential (TRP) (Fig. 3D-E). These studies have shown that when magnetic nanoparticles (MNPs) are exposed to a radiofrequency magnetic field (approximately 1–60 MHz), they can generate localized heat without causing significant overall heating [171]. This capability allows MNPs to act as precise triggers for thermally reactive molecules within mammalian cells. Specifically, structures sensitive to temperature changes, including those containing TRP or K2P channels, can transform these localized temperature shifts into cellular signals. One application of this technology is the local thermal activation of TRPV1 ion channels using 6 nm manganese ferrite nanoparticles, which has successfully induced action potentials in laboratory-grown neurons [41]. Moreover, thermally gated ion channels TRPV1 can be activated in response to the hysteretic heating of nearby magnetic nanoparticles when exposed to magnetic fields alternating at frequencies exceeding 100 kHz (Fig. 3D) [164]. Furthermore, magnetothermal deep brain stimulation (DBS) has been effectively applied to three different brain areas in mice, each associated with the regulation of distinct motor behaviors. This method involves the magnetic stimulation of neurons overexpressing TRPV1 in these areas, resulting in behaviors that directly correlate with the application of the magnetic field in the treated mice [78]. This technique of non-invasive in vivo regulation of neuronal activity has also been extended to include the expression of anti-ferritin nanobody-TRPV1 within targeted regions of the mouse brain [172]. Another similar approach for wireless DBS involves the thermal stimulation of TRPV1 channels using untargeted MNPs, which has proven effective in activating TRPV1-modified neurons within a specific region of the mouse brain [173]. Additionally, the regulation of adrenal hormone secretion through magnetothermal stimulation of TRPV1 channels has been demonstrated in mice that have not been genetically modified [173].

In contrast to conventional methods that employ a single type of MNP within a fixed magnetic field, recent studies have explored magnetothermal multiplexing. This technique involves selectively heating different MNP groups by varying the magnetic field’s amplitude and frequency, allowing for precise control over cell signaling. This was demonstrated in HEK293FT cells engineered to express TRPV1, highlighting the potential for fine-tuned cellular manipulation [174]. Furthermore, Sebesta et al. have shown that combining MNPs with variable magnetic field strengths and frequencies can induce quick behavioral responses in *Drosophila melanogaster* by activating the TRPA1-A rate-sensitive channel in the subsecond range [79].

Aside from these advancements, TRP channels have also been suggested to activate mechanically at lower field alteration frequencies. A notable study by Wheeler et al. involved a genetically encoded magnetic actuator created by fusing ferritin with the TRPV4 channel. This actuator was used to influence the behavior of zebrafish and mice moving freely under slow alternating magnetic fields [117]. However, the precise mechanism of TRPV channel activation in these ferritin-based experiments remains unclear [115, 116, 118]. In research conducted by Jonathan Dordick and colleagues, experiments on HEK cells and mice utilized the ferritin conjugated to the TRPV1 channel. These studies involved transfecting HEK cells with a system combining anti-GFP–TRPV1/GFP–ferritin and a calcium-responsive insulin gene construct. Following radiofrequency (RF) treatment, this setup led to enhanced calcium-dependent insulin gene expression. The application of this system successfully reduced blood glucose levels in vivo, either by implanting engineered stem cells or through the hepatic expression facilitated by a recombinant adenovirus [103]. Dordick et al. also applied this anti-GFP–TRPV1/GFP–ferritin system to remotely modulate blood glucose levels by targeting a subset of hypothalamic neurons sensitive to glucose [113]. This effect is thought to arise from the mechano-thermal activation of TRPV1 by ferritin within an oscillating or intermittent magnetic field. Nonetheless, an alternate explanation exists because the magnetic field-induced forces in this study were significantly lower than those typically required to activate mechanically sensitive ion channels [175]. Beyond these ferritin-based actuators, the use of magnetite nanodiscs in slowly varying magnetic fields has been shown to initiate a Ca^2+^ influx in mechanosensory neurons and to activate TRPV4 channels artificially expressed in HEK293 cells [176]. Moreover, it was demonstrated that TRPV4 channels can be activated through ferritin magnetocalorics (Fig. 3E) [142]. Additionally, an approach for wireless DBS that activates intrinsic Transient Receptor Potential Canonical (TRPC) channels using magnetic nanodiscs in a slow alternating magnetic field has been introduced [54].

The focus has also extended to the K2P family, particularly the TREK1 channel, which has been the subject of various magnetogenetic studies. Magnetic nanoparticles coated with anti-His antibodies have been utilized to activate TREK1 channels that have a His-tagged extracellular loop, resulting in alterations in whole-cell currents [177]. Magnetic Ion Channel Activation (MICA) technology has employed TREK1 intracellular loop antibodies to functionalize MNPs ranging from 250 nm to 1 μm, triggering Ca^2+^ influx through TREK1 in a 1 Hz oscillating magnetic field [178]. These approaches have indicated the potential of TREK1 activation in promoting bone regeneration and osteogenesis, with MICA facilitating collagen synthesis and mineralization by human mesenchymal stem cells in static magnetic fields [73]. Moreover, MICA has been shown to boost the expression of osteogenic markers through the use of TREK1 and Piezo1 functionalized graphene oxide-based nanocomposites [179]. Additionally, TREK1 activation via MICA has been applied in controlling neuronal cell signaling, initiating c-Myc/NF-*κ*B stress response pathways, and increasing neurite number in SH-SY5Y neuronal cell lines [180]. Lastly, the magnetothermal silencing of TREK1 has been explored to diminish dopaminergic reward responses in mice [181].

Thus, the magnetic activation of thermo- and mechanosensitive channels offers broad applications, largely due to the straightforward activation by magnetic nanoparticles and the diverse potential for cell state modulation. Magnetogenetics opens up new avenues for manipulating cellular structures beyond just ion channels, indicating a vast field of potential research and application.

### Magnetogenetic manipulation of cell junctions: bridging cellular mechanics and signaling

Cell junctions play a pivotal role in maintaining the structural integrity and signaling communication within tissues. E-cadherins, central to this network, are cell adhesion molecules crucial for forming adherens junctions, enabling cellular attachment through calcium-dependent mechanisms. These molecules are composed of an extracellular domain that binds to identical cadherin molecules on neighboring cells, and an intracellular domain that, along with adapter proteins like α, β-, and γ-catenins, links cadherins to the cytoskeleton’s actin filaments [182]. This intricate connection is fundamental in maintaining various physiological barriers, and its disruptions can lead to severe conditions, including inflammatory bowel disease [183], and oral pathogen-related diseases [184].

**Fig. 4:**
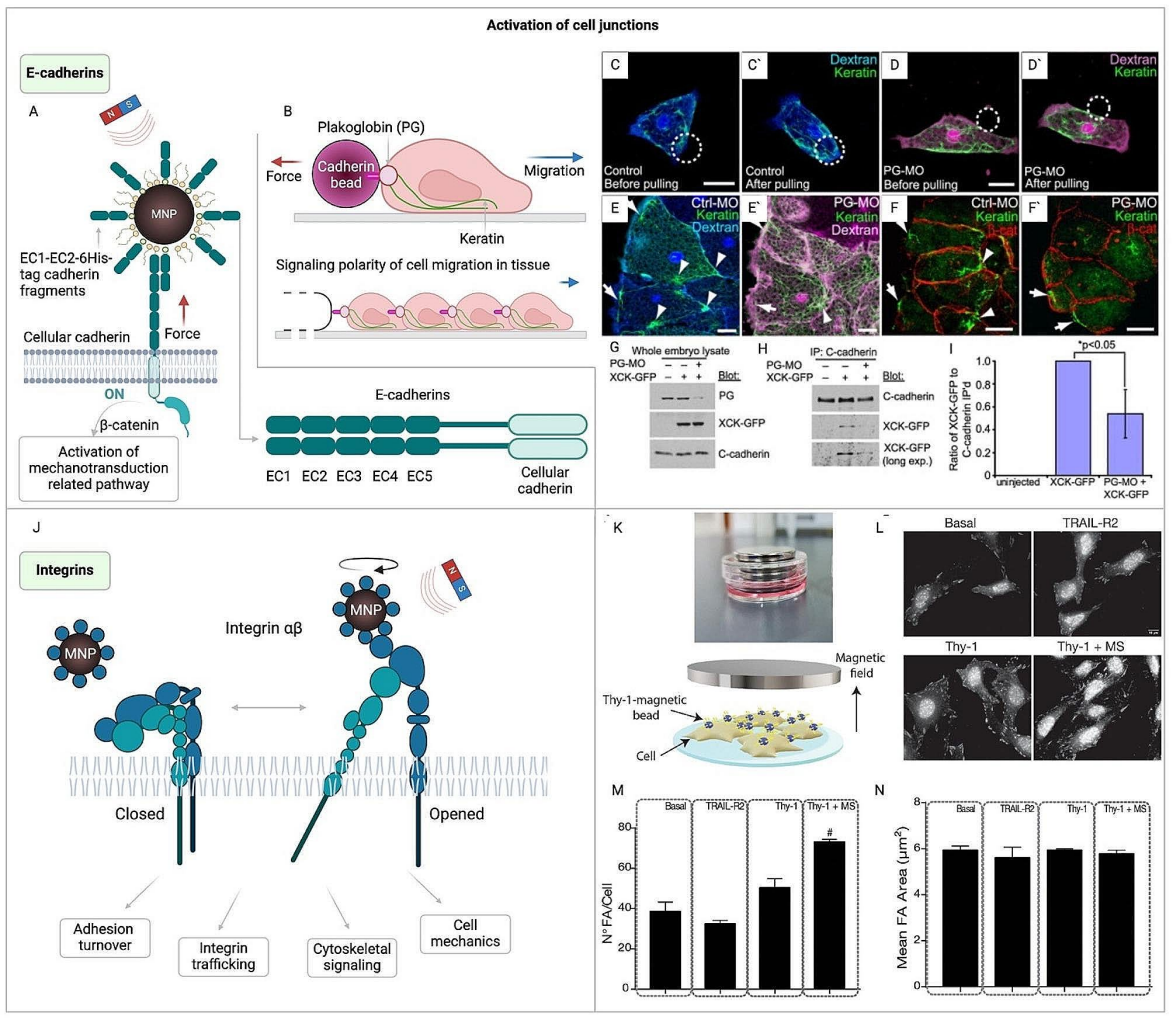
Application of magnetogenetics for activation of cell junction and cytoskeletal associated pathways. (**A**) Magneto-mechanical actuation via cadherin-nanoparticle bioconjugates. MNP – magnetic nanoparticles [185]. (**B**) Polarized cell behavior and migration directed by mechanoresponsive cadherin-keratin complex [186]. (**C-I**) PG necessity for Cadherin/Keratin Link. (**C, D**) Isolated cells marked with Alexa-dextran, showcasing GFP-XCK1(8) (green) expression and cultured on fibronectin. (**C**) and (C′) depict a standard cell (blue dextran), while (**D**) and (D′) display a PG-deficient cell (magenta dextran). C-cadFc bead (circle) attaches (**C** and **D**), then is pulled away (C′ and D′). (E, E’) Control (blue dextran) (**E**) and PG-deficient (magenta dextran) (E’) mesendoderm samples expressing GFP-XCK1(8) (green). (F, F’) Control (**F**) and PG-deficient (F’) mesendoderm in full embryos, stained for XCK1(8) (green) and β-catenin (red). (E–F’) Arrows indicate cabling at the leading-edge cells’ front, and arrowheads point to KIF clusters near intercellular junctions. All measurement bars represent 25 μm. (**G-I**) Embryos received injections of XCK1(8)-GFP, with or sans PG morpholino. (**G**) Embryo extract immunoblots reveal XCK1(8)-GFP and innate PG levels, with or without PG morpholino (PG-MO). (**H**) Immunoblots of C-cadherin co-precipitates for XCK1(8)-GFP and C-cadherin, with or without PG-MO. (**I**) Three separate co-immunoprecipitation experiments quantified, presented as average ± SEM [186]. (**B-I**) reprinted from [186]. © 2011 Cell Press (Open Access). (J) Activation and regulation of Integrin by MNP [187–189]. (**K-N**) Indirect immunofluorescence analysis of focal adhesions in DITNC1 cells. (**K**) The top image displays the setup with magnets below the tissue culture plate, generating a magnetic field. This diagram details the experimental setup: Cells were seeded on coverslips (light blue) and treated with serum-free medium for 30 min. DITNC1 cells (beige) were then exposed to either TRAIL-R2- (as a control) or Thy-1-coated Protein A magnetic beads (blue balls with yellow projections), with or without mechanical stress (MS) induced by a magnet (gray) for 5 min. (**L**) Focal Adhesions (FA) were identified using an antibody for phospho-tyrosine and a secondary antibody. Scale bar = 10 μm. (**M, N**) The data in the graph represent average + s.e.m. for at least 30 cells under each condition in three separate experiments (*n* = 3), showing the count (N°) of FAs per cell (**M**) and the mean area (µm²) of the FAs (**N**). Statistical significance was assessed using the Kruskal–Wallis non-parametric test followed by Dunn’s multiple comparisons test. #*p* < 0.05, indicating significance against the Thy-1 condition [190]. (K-N) reprinted from [190]. © 2021 Frontiers (Open Access)

The role of intercellular force recognition is crucial in maintaining tissue integrity and facilitating cell signaling. When cells experience mechanical force, it leads to the activation of E-cadherin, which promotes intracellular stabilization of F-actin and recruitment of vinculin for enhanced junctional stability [191]. These findings support the idea that cadherin-mediated intercellular junctions control the cellular contractile machinery in a mechanosensitive manner. Given their direct linkage to the cytoskeleton, these receptors are prime for mediating mechanochemical signal transduction. Utilizing magnetic nanoparticles to target cadherin molecules allows for direct mechanical manipulation (Fig. 4A). Techniques like magnetic tweezers, which pull on superparamagnetic beads attached to C-cadherin’s extracellular domain, have illustrated how cadherin-based forces can regulate cell polarity and encourage collective movement (Fig. 4B-I) [186]. In addition, this method has revealed that both homotypic and heterotypic cadherin junctions can endure similar forces, with heterotypic junctions eliciting a mechano-sensitive reaction in cancer cells, showcasing the intricate balance between cellular attachment and mechanotransduction in health and disease [192].

Furthermore, cadherins are crucial for the maintenance and rearrangement of endothelial junctions, which play roles in diverse biological processes, from leukocyte migration and wound healing to tumor invasion and the development of atherosclerosis. Beyond the effects of mechanical tension and actin polymerization on cadherin activation, signaling pathways such as RhoA and Rac1 have been identified as regulators of adherens junctions [193]. Magnetogenetic techniques were utilized to precisely control the location and timing of intracellular signaling molecule activation, particularly targeting Rho-GTPases signaling pathways. Through the use of magnetically functionalized nanoparticles, which acted as points of activation that could be moved by a magnetic field, it was possible to initiate signaling pathways. This led to the reorganization of the actin cytoskeleton and subsequent changes in cell morphology, showcasing the potential of magnetogenetics in modulating cell behavior and tissue dynamics [92].

Integrins represent another crucial component, linking the external mechanical environment to the cell’s internal cytoskeleton and facilitating mechanosensation (Fig. 4J) [187–189]. The ability of cells to sense their mechanical surroundings via integrins impacts numerous cellular pathways, with significant mechanosensitive behaviors driven by YAP/TAZ requiring integrin-mediated adhesion for activation [92, 194, 195]. Specifically, integrin signaling plays a pivotal role in controlling cell growth, and in the progression and invasion of tumors [196–198], as well as in fibrosis and the migration and functional regulation of immune cells [199–201], and function of neurons (Fig. 4K-N) [190]. Building on this understanding of integrins’ pivotal role in mechanosensation and cellular response, magnetogenetics emerges as a powerful tool. It offers a method to manipulate integrins on the cell membrane, enabling the mediation of mechanical tension through the application of a constant or slowly alternating magnetic field to magnetic beads coated with integrin antibodies. This technique, employing beads approximately 4.5 μm in diameter, allows for precise application of force to study the kinetics and likelihood of neurite initiation [189, 199], test fundamental mechanotransduction hypotheses [202] or cellular adaptation to mechanical stress [203], and demonstrate the role of integrins in collagen expression regulation in tendon cells [204]. Additionally, smaller MNPs, sized between 250 nm and 1 μm and coated with integrin antibodies, have been used to activate the ERK pathway in HEK293 cells via the MICA system [178]. Furthermore, a therapeutic approach using iron oxide nanoparticles in a constant magnetic field has been proposed for osteoporosis treatment, targeting integrin expression [188].

Thereby, through the magnetogenetic manipulation of both cadherins and integrins, a variety of signaling pathways, including ERK, YAP/TAZ, and RHO, can be activated. These pathways play a significant role in controlling a range of biological processes, from cell migration and growth to differentiation, showcasing the broad applicability and potential of magnetogenetics in biological research and therapy.

### Utilizing magnetogenetics to explore cytoskeletal dynamics and mechanotransduction

Most of our understanding of how a cytoskeleton responds to mechanical deformation is based on studies investigating compression-stretching of the entire cell. This broad deformation activates cytoskeleton-associated mechanosensitive signaling pathways [205]. However, the role of individual components of the cytoskeleton (actin filaments, intermediate filaments, microtubules) in the transmission of mechanosensitive signaling pathways remains unclear [206]. There are different ways to study the cytoskeleton and associated signaling. However, most of these methods involve indirect techniques to regulate the polymerization and depolymerization of cytoskeletal components [207, 208]. Furthermore, the mechanical rearrangement of individual elements of the cytoskeleton can activate various elements of mechanosensitive signaling. To address this, MNPs can be used to “pull” individual elements of the cytoskeleton and activate the mechanosensitive signaling.

**Fig. 5:**
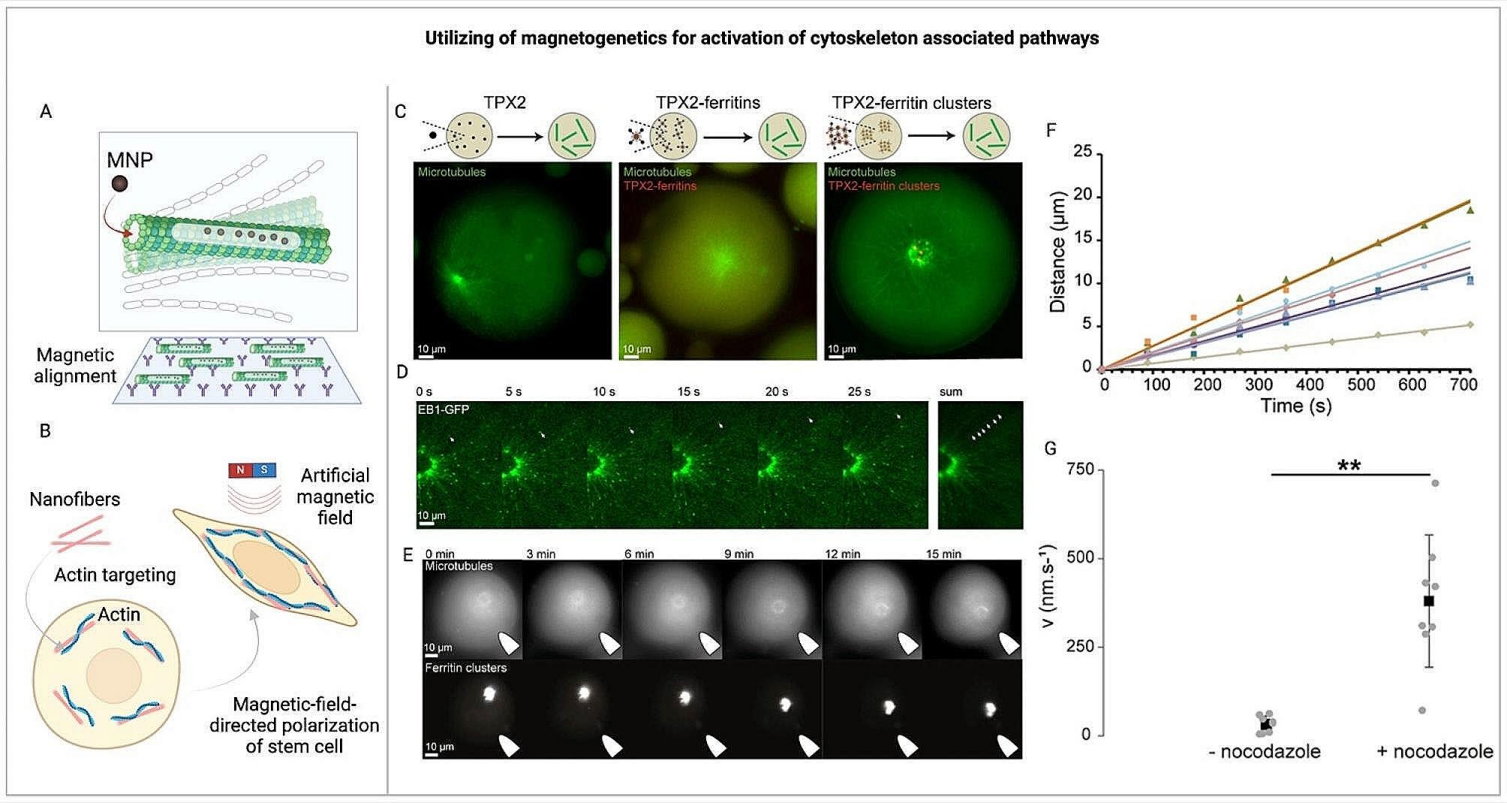
Utilizing of magnetogenetics for activation of cytoskeleton associated pathways. (**A**) Magnetic alignment of microtubules with CoPt nanoparticle-encapsulating Tau peptides [209]. (**B**) Magnetic-field-guided polarization of stem cells via supramolecular nanofibers [72]. (**C-G**): Initiation and precise control over the formation and movement of microtubule structures using magnetic fields. (**C**) Using confocal microscopy, the formation of microtubules was observed in *Xenopus* egg extract droplets, initiated by FKBP-TPX2. These microtubules and ferritins were marked with fluorescein-tagged tubulin and mCherry, respectively. Sequentially, the formation of microtubule networks was induced by FKBP-TPX2, followed by mCherry/TPX2-ferritins, and then ferritin-TPX2 aggregates. (**D**) A sequence of images showing the expansion of microtubules from a central point, known as an aster, prompted by clusters of ferritin. EB1-GFP served as a marker for the growing ends of the microtubules. (**E**) Selected moments capturing the movements of an aster’s center driven by a magnetic field. (**F**) Illustration of how aster centers move along a magnetic field gradient. This is shown by a graph depicting the path length of an aster over time under the influence of magnetic forces. (**G**) The average speed of asters moving towards a magnet, measured with and without the application of a microtubule-disassembling agent (nocodazole) [210]. (**C-G**) reprinted from [210]. © 2017 Springer Nature (Open Access). FKBP – FK506 binding protein; TPX2 – targeting protein for Xklp2; EB1 – end binding protein 1; GFP - green fluorescent protein

Transitioning from these foundational insights, it’s important to acknowledge the challenges and opportunities presented by magnetogenetics in this field. Despite the frequent observation of nonspecific cytoskeletal rearrangement or filament disruption within magnetogenetics, largely as a result of widespread effects from mechanical [208, 211] and thermal perturbations [212], the technique holds potential for targeted manipulation. In vitro experiments utilizing nanoparticles and external magnetic fields have successfully demonstrated mechanical reconfigurations of cytoskeletal components, specifically with artificially polymerized actin and tubulin (Fig. 5A, B) [72, 209, 213, 214]. For example, Chen et al. explored cytoskeletal manipulation through the attachment of superparamagnetic iron oxide particles to biotinylated g-actin. By applying a uniform magnetic field, they were able to influence actin’s alignment, polymerization, and its movement over myosin [68]. Similarly, ferritin-TPX2 scaffolds have been employed to control the assembly of microtubules in a manner akin to centrosome organization [210]. Nonetheless, comprehensive strategies for rearranging the cytoskeleton within cells, particularly for directing actin filament polymerization, remain scarce (Fig. 5C-G) [210]. This gap highlights the potential of magnetogenetics to pioneer new investigations into intracellular signaling pathways, including those intertwined with cytoskeletal dynamics.

## Ligand-free induction of ligand-mediated signaling through magnetogenetics

Transmembrane cell surface receptors are key mediators in transmitting extracellular signals to the cell’s interior, bridging the external environment with internal biochemical pathways. These receptors typically feature a transmembrane domain (TMD) that links an extracellular domain (ECD) to an intracellular domain (ICD), responding to external stimuli via a single transmembrane alpha helix and conveying information to intracellular effector proteins. Investigating these receptors is crucial as they play a central role in numerous physiological processes and disease mechanisms, making them targets for therapeutic intervention [215–217]. Magnetogenetics presents a unique tool for this exploration, offering a way to activate these receptors in a ligand-free manner and study their functions and the resulting cellular responses in real-time. Utilizing MNPs and magnetic fields, researchers have manipulated cell activity by targeting these membrane receptors with subsequent rotation, clustering, or movement, effectively activating the receptors without traditional ligand binding. Achieving binding specificity often involves coating the particles with antibodies or specific ligands targeted at membrane proteins [78]. This innovative approach has demonstrated the potential of magnetic forces, mediated by MNPs, to activate various signaling pathways, including Ca^2+^ signaling, Src family protein kinases, MAPK, Wnt, Notch receptors, and RhoGTPase pathways. Such versatility highlights the expansive utility of magnetogenetics in cell signaling research and potential therapeutic applications [57].

### Exploring ligand-free receptor dimerization and clustering via magnetogenetics

**Fig. 6:**
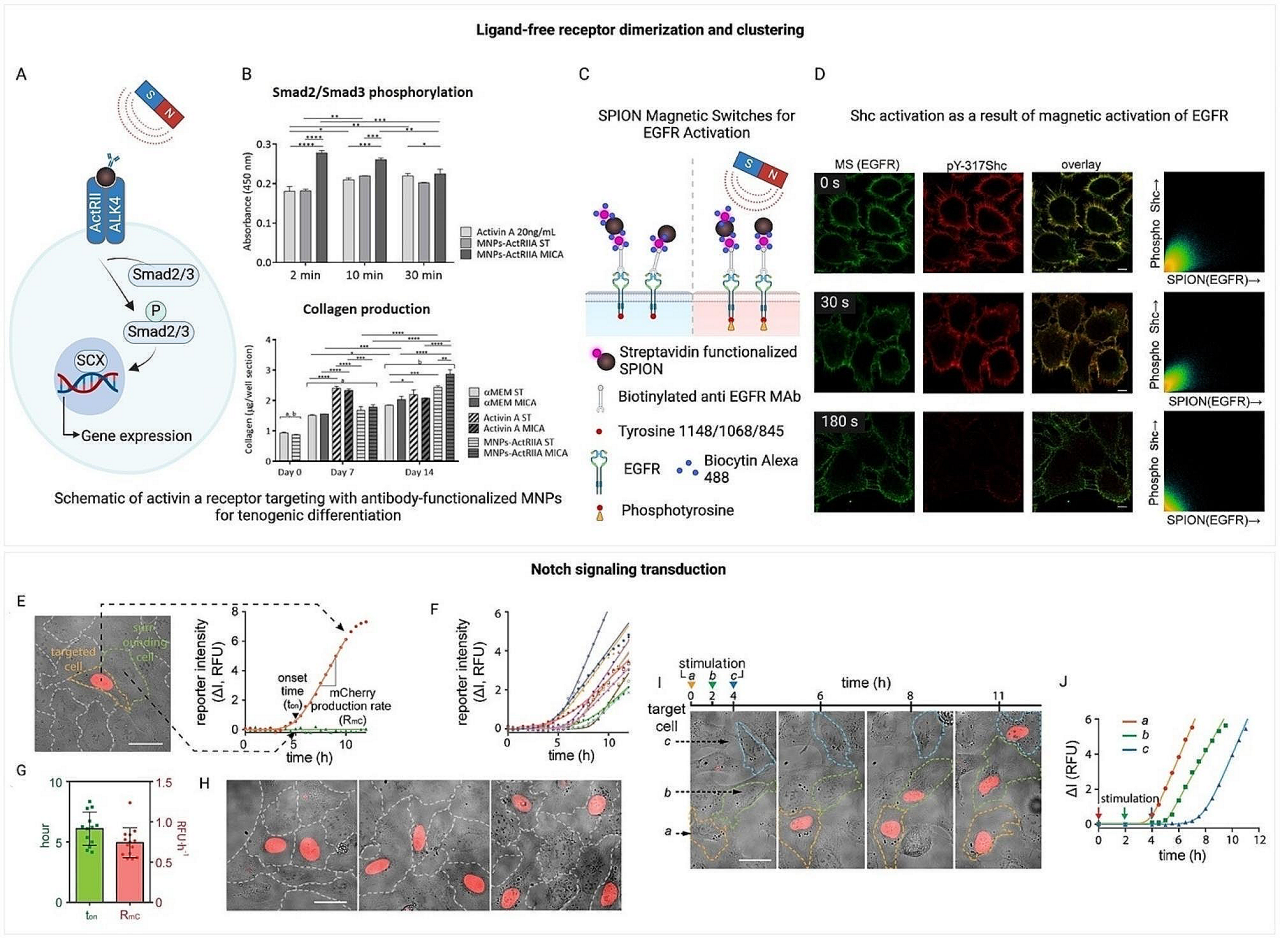
Ligand-free induction of ligand-mediated signaling. (**A-B**) Ligand-free receptor dimerization and clustering [51]. (**A**) The diagram depicts the process of labeling human adipose-derived stem cells (hASCs) with magnetic nanoparticles (MNPs) targeting the activin A receptor (ActRIIA). Upon stimulation with a magnetic field, the labeled cells activate the intracellular signaling pathway, specifically TGF-β/Smad2/3, leading to the promotion of tenogenesis. (**B**) Smad2/Smad3 Phosphorylation: The first chart on the right top illustrates the phosphorylation level of Smad2/Smad3 over time, following stimulation with activin A and two different types of MNP complexes targeted to the activin A type IIA receptor (ActRIIA). Collagen Production: The second chart on the right bottom shows collagen production in cell cultures measured at day 0–14. The quantification is based on Sirius Red staining, which binds to collagen fibers. The graph compares collagen levels across different culture conditions, including control groups, activin A-treated groups, and MNP-ActRIIA complex-treated groups, demonstrating how collagen production changes over the culture period and under different stimulatory conditions. Reprinted with permission from [51]. © 2018 Elsevier Inc. (**C-D**) SPION magnetic switches for EGFR activation (**C**) and Shc activation as a result of magnetic activation of EGFR (**D**). Confocal immunofluorescence displays cells post 15-minute incubation at 37 °C subsequent to 0–180 s (0, 30, 180 s) of magnetic field application. From left to right, the columns show: MS Alexa-488 biocytin labeling (green); indirect immunofluorescence with monoclonal antibody specific to pY-317 Shc protein paired with GARIG-CY5 (red); a composite of the first two columns; two-dimensional colocalization scatter plots of magnetic switches (MS) and pY317-Shc fluorescence signals after refining 50 optical slices with SVI Huygens software [56]. (**C-D**) reprinted from [56]. © 2013 PLOS One (Open Access). (**E-J**) Spatial and temporal control of Notch signaling transduction with MPNs. (E–J) Dynamics of H2B-mCherry Expression in UAS-Gal4 Reporter Cells Following MPN-Triggered Notch Signaling. (**E**) Showcases a typical fluorescence image alongside a temporal data graph. (**F**) Compilation of time series data from multiple cells. (**G**) Evaluates the initiation timing (ton) and production speed (RmC) of mCherry. (**H**) Deliberate spatial activation of Notch signaling. (I and J) Regulation over time of Notch pathway activity. (I) Sequential images over time and (J) tracks of mCherry fluorescence intensity for three randomly selected cells (labeled a, b, and c) within a group, following staggered stimulations at 2-hour intervals [57]. (E-J) reprinted from [57]. © 2016 Cell Press (Open Access)

Exploring receptor signaling without traditional ligand binding, magnetogenetics could potentially facilitate a deeper understanding of how receptors like the epidermal growth factor receptor (EGFR) within the ErbB protein family [218], receptor tyrosine kinases (RTKs) [219], Toll-like receptors [220], cytokine receptors, and the erythropoietin receptor (EpoR) [221] are activated. This approach reveals that receptor dimerization, triggered by ligand binding [222], is a critical step for aligning intracellular domains, enabling cross-phosphorylation and the initiation of downstream signaling (Fig. 6A-B) [51, 223, 224]. Notably, while certain receptors may exist in inactive dimeric forms—such as EpoR [225], EGFR [221], epinephrine receptor EphA2 [226], and the insulin receptor [227]—their activation is often contingent upon clustering at high membrane densities [228], a process naturally facilitated by ligand-induced dimerization. This dimerization process not only aligns intracellular domains but also causes significant structural rearrangements within the receptor itself, including translation, piston, pivot, and rotation of the transmembrane domain [229], critical for disengaging the kinase domain from autoinhibition and achieving an active receptor configuration.

The ability of magnetogenetics to manipulate these processes marks a significant leap in receptor signaling research. By bypassing traditional ligand-receptor interactions, magnetogenetics enables precise receptor activation and unlocking insights into receptor signaling, thereby facilitating an exploration of cellular communication and signaling dynamics in health and disease. Specifically, certain members of the family of RTKs, including PDGFR, TGFb and EGFR, can be activated through the use of magnetic nanoparticles by clustering on the cell membrane, phosphorylation of the receptor, and activation of secondary signaling pathways (Fig. 6C-D) [51, 56, 74, 230]. This process alters gene expression patterns typically associated with cell differentiation, demonstrating the technique’s potential for precise cellular control.

Building on this foundation, magnetogenetic receptor clustering has been successfully applied in immunology and cancer therapy, particularly in manipulating death receptors, TNF, and T cell receptors. For example, zinc-doped iron oxide MNPs targeted at death receptor 4 have induced apoptosis in DLD-1 colon cancer cells [45], showcasing the method’s effectiveness beyond the petri dish. This approach has also yielded promising results in vivo, such as inducing apoptosis in zebrafish by clustering ovarian TNF receptors [45]. Furthermore, the use of magnetogenetics for T cell receptor clustering with antigen-presenting magnetic particles has significantly enhanced T cell expansion in vitro [231] and, when applied in vivo, has shown efficacy in inhibiting B16 melanoma growth in mice [231], highlighting magnetogenetics’ expanding role in therapeutic interventions.

### Magnetogenetic activation of notch signaling pathways

Notch signaling plays a pivotal role in regulating cell proliferation, death, and differentiation, critical for tissue development and maintenance. This signaling pathway is activated when the Notch receptor binds to its ligand, leading to receptor clustering and subsequent conformational changes. These changes release the Notch intracellular domain (NICD) [232]. This domain then travels to the nucleus, where it joins other proteins to influence the expression of critical genes involved in cell growth and apoptosis, such as Myc, p21, and cyclin D3 [233]. Interestingly, research has shown that mechanical forces alone can activate the Notch receptor (Fig. 6E-J) [57]. Specifically, the unfolding of the Notch protein’s negative regulatory region (NRR) by mechanical stress exposes a crucial site for cleavage by metalloproteinases. This exposure facilitates the release and nuclear migration of the NICD, highlighting a direct pathway for activation independent of ligand binding [57, 234].

Moreover, the force of ligand binding required to stimulate Notch1 receptors falls between 1 and 20 pN [235], contrasted with the stronger interaction forces observed between integrins and fibronectin, around 50 pN [236]. Employing magnetic tweezers and nanoparticles, researchers have applied pulling forces as minimal as 9 pN to the Notch receptor [237]. This method efficiently separates its extracellular domain, consequently activating the Notch signaling pathway [57]. Thus, the utilization of magnetogenetics in probing and modulating Notch signaling not only enriches our understanding of cellular mechanisms but also opens avenues for developing therapeutic interventions for conditions associated with Notch signaling anomalies.

### Magnetogenetic manipulation of G protein-coupled receptors (GPCRs)

**Fig. 7:**
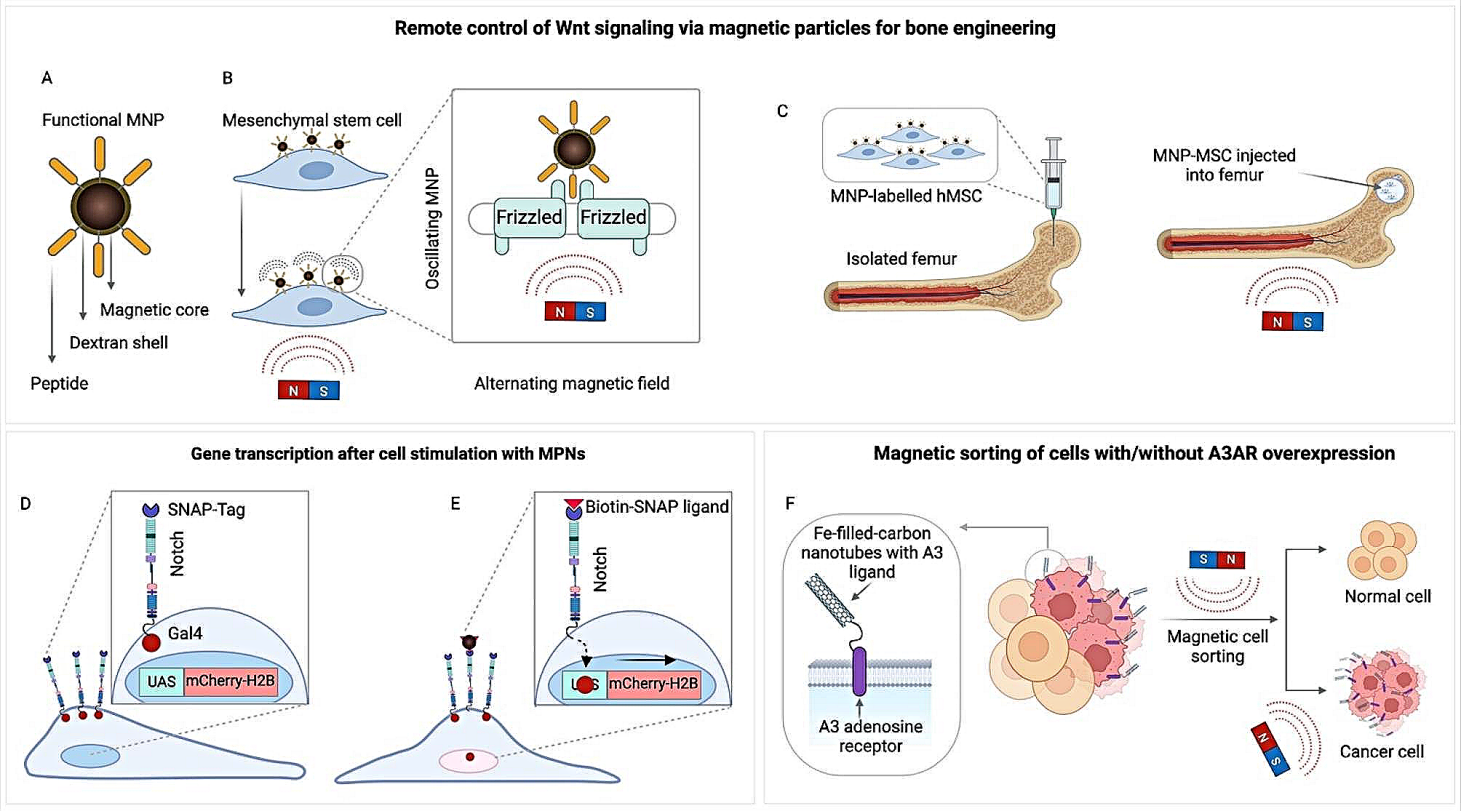
**G protein-coupled receptors activation (GPCR family).** (**A-C**) A schematic representation of the use of MNP and the sequential steps of cell labeling and stimulation or cell injection into femurs targeting Wnt signaling. Initially, MNPs are tailored with specific binding agents like peptides (**A**). Subsequently, cells are marked with these bespoke MNPs. Post-labeling, the cells can either be exposed to fluctuating magnetic fields in a laboratory setting to trigger receptor aggregation and the commencement of signaling pathways (**B**), or they can be introduced as a cell-MNP mixture into tissue engineering constructs, such as a fetal chick femur, and then magnetically manipulated to modulate bone development in an ex vivo environment (**C**) [251]. (**D-E**) Activation of Notch signaling by mechanical force. (**D**) A graphical depiction of the live-cell assay designed to observe the activation of the Notch receptor triggered by mechanical stress. The engineered receptor is linked to streptavidin-coated paramagnetic beads via a SNAP-biotin connector. (**E**) Application of mechanical force across the receptor results in the severance of its C-terminal domain. In this particular setup, the C-terminal domain has been substituted with Gal4 to serve as a transcriptional indicator, which in turn promotes the synthesis of mCherry-H2B [244]. (**F**) Magnetic sorting of cells with/without A3AR overexpression. Ligands for the A3 adenosine receptor attached to iron-filled carbon nanotubes were created to specifically target certain cancer cell types for magnetic cell separation and thermal treatment in cancer therapy. Although these nanostructures could effectively attach to the A3 adenosine receptors, they failed to demonstrate selective binding to cells that overexpressed the receptor upon cellular interaction [245]

G protein-coupled receptors (GPCRs), the most extensive family of cell surface receptors, play a crucial role in mediating cellular responses to diverse signals such as hormones, light, and neurotransmitters, and they act as mechanosensors triggered by mechanical forces (Fig. 7A-F) [57]. This sensitivity to mechanical stimulation introduces a novel trigger for activating various GPCR-associated signaling pathways [238], including those involved in vascular autoregulation where mechanical stress from high blood pressure activates mechanosensitive GPCRs like the angiotensin II type 1 receptor (AII1R) [239], histamine H1 receptors (H1Rs) [240], GPR68 [241], dopamine receptor type 5 (DR5) [242], leukotriene receptor CysLT1R [243], Notch signaling (Fig. 7D-E) [244], different receptors such as A3 adenosine receptor (A3AR) (Fig. 7F) [245], and other, leading to vasodilation. The mechanism underlying the action of mechanosensitive adhesive GPCR proteins involves an autoproteolytic reaction within the GAIN domain, resulting in the receptor splitting into two fragments that non-covalently assemble on the cell surface, providing a molecular basis for mechanical force sensing [246, 247]. Ligand binding induces a conformational change in GPCRs, activating Gα-subunits and downstream signaling pathways, influencing various cellular processes through adenylyl cyclase activation, cAMP level alterations, or phospholipase C activation [248–250]. Investigating these receptors and signaling pathways is essential due to their central role in physiological and pathological processes, with magnetogenetics emerging as a powerful tool to facilitate this research by enabling the precise control of receptor activation in a ligand-free manner, thus offering new insights into the complex interplay of signaling pathways in health and disease.

Building upon the basis of magnetogenetics, the use of magnetic tweezers and beads has played a crucial role in proving that mechanical forces can trigger the separation of the GAIN domain within adhesive GPCRs, paving the way for novel techniques to regulate cellular functions [252, 253]. This revelation is particularly significant as it highlights how mechanical forces can influence receptors previously considered insensitive to such stimuli. The introduction of post-translational modifications and alternative splicing within the GPCR’s intracellular domain at the C-terminus gives these receptors mechanosensing abilities [254, 255], which suggests that mechanical forces – possibly in combination with hormonal signals such as PTH (1–34) – can trigger a response independent of traditional ligand binding [256].

Expanding upon these insights, magnetogenetics’ capacity to direct cell fate has been further demonstrated in studies involving mesenchymal stem cells (MSCs) equipped with superparamagnetic iron oxide nanoparticles aimed at the Wnt receptor Frizzled (Fig. 7A-C). These studies revealed that pulsed magnetic fields could drive osteogenic [251] and neuronal differentiation [257], instigating Wnt signaling [258] and β-catenin translocation [251, 259]. This finding underscores magnetogenetics’ capability to navigate cell destiny. Moreover, the application of mechanical stimuli has been shown to trigger specific signaling pathways, such as those activated by β2-adrenergic receptors under traction forces [255], and G proteins (Gαq/11) engaged in mechanotransduction beyond conventional GPCR activation [260]. Targeting G protein-coupled receptor 91 (GPR91)/STAT3/VEGF pathways with superparamagnetic iron oxide nanoparticles modified with miR-326 exemplified magnetogenetics’ precision in curtailing tumor proliferation through distinct signaling pathways [261], highlighting its therapeutic promise.

Together, these findings shed light on the vast potential of magnetogenetics in investigating and controlling the intricate network of cellular signaling pathways. This presents new and promising opportunities for research and therapeutic advancements. The growing field of magnetogenetics, particularly in the precise activation of GPCRs using magnetic nanoparticles, marks an exciting frontier in cellular manipulation.

## Conclusion and future perspectives

Magnetogenetics, a field that primarily utilizes artificial superparamagnetic or ferromagnetic nanoparticles, has made remarkable progress in locally generating heat and mechanical forces with nanometer-scale spatial and microsecond-scale temporal precision. However, despite significant progress, the field still faces challenges, including the need for invasive nanoparticle injections that may risk tissue damage and have low targeting efficiency. To address these obstacles, the exploration of genetically programmed magnetic nanoparticles or electromagnetic sensing proteins is currently explored as a promising solution. This approach may potentially overcome these challenges and mark the advent of a new phase in the development of magnetogenetic systems.

A breakthrough came with the discovery of the Electromagnetic sensing gene (EPG) from *Kryptopterus bicirrhis*, coding for a membrane protein responsive to electromagnetic fields. Successfully cloned and expressed in mammalian cells, neuronal cultures, and the rat brain, EPG has demonstrated potential in various applications. For instance, its remote activation by electromagnetic fields (EMF) can significantly boost intracellular calcium levels in both mammalian cells and cultured neurons, signaling enhanced cellular excitability. Furthermore, activating EPG wirelessly in the rat motor cortex has elicited motor responses in the contralateral forelimb, demonstrating its potential in vivo. These findings suggest that EPG’s activation, especially when targeted to specific neural populations like inhibitory interneurons, could suppress seizure activities, offering a novel, cell-specific, and closed-loop approach to mitigating seizure severity [262, 263].

Despite the groundbreaking potential of EPG, naturally occurring proteins that respond to EMF are exceedingly rare. An alternative solution could be biological magnetic nanoparticles called magnetosomes, which are also infrequently found in nature and in a specific group of bacteria capable of producing them [264, 265]. Leveraging genes responsible for the biomineralization of magnetite offers a pathway to imbue human cells with magnetic sensitivity, mirroring strategies used in optogenetics. Through viral vectors, these genes could be specifically delivered to certain types of cells, paving the way for magnetically responsive human tissues in the future. Critical to this approach is understanding the mechanism behind magnetosome formation, which is orchestrated by key magnetotactic proteins. These proteins create an environment within magnetosome vesicles characterized by high pH and low redox potential conducive to the nucleation and growth of magnetic nanoparticles. Some proteins, such as Mms7 (also known as MamD), are thought to serve as templates shaping the crystal lattice’s spatial arrangement, while others like Mms5 and Mms6 influence crystal growth by interfacing with the crystal surface [266–268]. Despite these mechanisms being well-established in bacteria, human cells lack the capacity to naturally form magnetosomes or respond to magnetic fields, underscoring a significant hurdle in applying magnetogenetics directly to human biology.

Nevertheless, the genetic engineering of mesenchymal stem cells to express magnetite-forming genes, such as the codon-optimized mmsF and mms6, has yielded promising outcomes, producing cells containing intracellular superparamagnetic nanoparticles ranging from 10 to 500 nm in size [107]. Additionally, exposing human mesenchymal stem cells to non-magnetic ferric salts has unexpectedly led to the biomineralization of iron. This process, observed over 21 days in human stem cells and 14 days in mice, leads to the accumulation of magnetic iron nanoparticles within the cell cytoplasm. It involves a transformation from Fe^2+^ to Fe^3+^ ions, culminating in the formation of ferrihydrite, alongside a minor magnetic phase. The implications of such biomineralization on cell viability and physiological stability are profound, warranting further exploration [269].

Bridging the gap between our current understanding and the vast potential of future innovations, magnetogenetics stands out as a groundbreaking force in both scientific research and medical practice. This advanced technique marks a significant shift towards a more dynamic control over cellular behavior and the modulation of physiological processes. At the forefront of this field, magnetogenetics offers promising possibilities, particularly in the context of regenerative medicine. Several studies have shown that utilizing the thermal or mechanical effects of magnetogenetics in stem cells can promote cell differentiation [51, 52] and tissue formation [53]. Specifically, this approach is promising in bone regeneration, where magnetogenetic activation of human MSCs through thermo- or mechanosensitive ion channels can drive osteogenic differentiation [251], facilitate collagen synthesis and mineralization [73], and boost the expression of osteogenic markers [179]. By employing magnetically responsive human stem cells, magnetogenetics introduces an innovative strategy for steering biological processes within the body. This approach enables the accurate transformation of cells into designated tissue types, thereby supporting precise and targeted tissue regeneration efforts.

Furthermore, the application of magnetogenetics extends into the domain of neural tissues, showcasing remarkable potential. The magnetic activation of neuronal differentiation in MSCs through the Wnt receptor Frizzled [257], the induction of action potentials in laboratory-grown neurons [41], the increase in neurite number in vitro, and neuromodulation in freely moving animals [59] all demonstrate magnetogenetics’ applicability to the field of neuroscience. The capacity to navigate nerve development and regulate neuronal activity with magnetic fields opens new horizons for treating a wide array of nervous system disorders, promising a future where controlling cellular activity could lead to groundbreaking therapeutic solutions.

Magnetogenetics also holds significant promise in cancer therapy. Previous research has demonstrated that magnetic nanoparticles can be engineered to specifically target and induce apoptosis in cancer cells through localized thermal or mechanical stimuli. For example, zinc-doped iron oxide MNPs targeted at death receptor 4 have induced apoptosis in DLD-1 colon cancer cells, showcasing effectiveness beyond the petri dish [45]. Additionally, the use of magnetogenetics has shown promising in vivo results, such as inducing apoptosis in zebrafish by clustering ovarian TNF receptors [45]. The use of antigen-presenting magnetic particles for T cell receptor clustering has significantly enhanced T cell expansion in vitro and inhibited B16 melanoma growth in mice [231]. This targeted approach provides a more precise and less invasive treatment option compared to traditional therapies. Furthermore, magnetic actuators have been applied to mechanically influence cell fate for cancer therapy [44–48], and high-frequency magnetic fields have been used to heat MNPs, activating thermosensitive ion channels and initiating heat-responsive promoters for cancer therapy [49]. This ability to modulate signalling pathways in cancer cells opens new avenues for developing novel therapeutic strategies.

Transitioning from regenerative and cancer medicine to more targeted therapeutic interventions, magnetogenetics also unveils new possibilities for managing cellular functions crucial to our health. A prime example is the development of magnetically sensitive insulin-producing cells, a revolutionary method for diabetes management [103]. These innovative cells, which can be implanted subcutaneously within a capsule, may be activated by magnetic fields to adjust proinsulin release directly into the bloodstream [103]. This ability to manipulate intracellular signaling pathways extends magnetogenetics’ reach, allowing for the meticulous regulation of metabolic processes and offering a novel avenue for tackling metabolic disorders, including obesity.

To conclude, magnetogenetics offers an immense opportunity to advance our knowledge of cellular behavior and lead the way for new medical interventions. The integration of magnetic field-sensitive cells into biomedical research and clinical applications is a promising frontier, but it requires careful scientific exploration and validation. This cautious approach will ensure a deeper comprehension of cellular mechanisms and pave the way for innovative treatments, positioning magnetogenetics as a valuable tool for enhancing patient care, within the bounds of research and ethical considerations. As we navigate this complex landscape, integrating magnetic field-sensitive cells into research and clinical practices will be a critical challenge in biomedicine. It not only deepens our grasp of cellular functions but also opens transformative possibilities for treating a broad spectrum of diseases and conditions through the controlled manipulation of cellular activity. This cautious yet hopeful path towards practical and beneficial future applications holds significant promise.

## Data Availability

Data will be made available on request.
